# Precise quantification of silica and ceria nanoparticle uptake revealed by 3D fluorescence microscopy

**DOI:** 10.3762/bjnano.5.173

**Published:** 2014-09-23

**Authors:** Adriano A Torrano, Christoph Bräuchle

**Affiliations:** 1 Department of Chemistry and Center for NanoScience (CeNS), University of Munich (LMU), Butenandtstrasse 5-13(E), 81377 Munich, Germany

**Keywords:** ceria nanoparticles, fluorescence microscopy, image analysis, nanotoxicology, silica nanoparticles

## Abstract

Particle_in_Cell-3D is a powerful method to quantify the cellular uptake of nanoparticles. It combines the advantages of confocal fluorescence microscopy with fast and precise semi-automatic image analysis. In this work we present how this method was applied to investigate the impact of 310 nm silica nanoparticles on human vascular endothelial cells (HUVEC) in comparison to a cancer cell line derived from the cervix carcinoma (HeLa). The absolute number of intracellular silica nanoparticles within the first 24 h was determined and shown to be cell type-dependent. As a second case study, Particle_in_Cell-3D was used to assess the uptake kinetics of 8 nm and 30 nm ceria nanoparticles interacting with human microvascular endothelial cells (HMEC-1). These small nanoparticles formed agglomerates in biological medium, and the particles that were in effective contact with cells had a mean diameter of 417 nm and 316 nm, respectively. A significant particle size-dependent effect was observed after 48 h of interaction, and the number of intracellular particles was more than four times larger for the 316 nm agglomerates. Interestingly, our results show that for both particle sizes there is a maximum dose of intracellular nanoparticles at about 24 h. One of the causes for such an interesting and unusual uptake behavior could be cell division.

## Introduction

Measuring the interaction between nanoparticles and cells is a mandatory step for the investigation of nanoparticles designed for medical treatment, and also for a correct risk assessment of nanoparticles. In both cases, knowledge regarding the kinetics of particle internalization gives the dose as a function of the time and allows for the investigation of a variety of parameters on that might influence the uptake behavior. Typical examples are particle characteristics such as size, morphology, chemical composition, surface charge and functionalization [1–3]. In addition, access to the number of intracellular particles is essential in studies aimed to compare the effect of similar particles on different cell types [4]. What all these investigations have in common, though, is the need for a fast and accurate method to quantify the uptake of nanoparticle by cells. In vitro cell culture experiments are well-known models to study the uptake of nanoparticles into human cells. Basically, a monolayer of cells is grown on the bottom of a culture well and nanoparticles are added to this culture to interact with the cells.

Fluorescence microscopy is commonly the method of choice to visualize this interaction because it can be performed on live cells with high spatial and temporal resolution. Finally, outcomes of the uptake process are normally assessed via qualitative and semi-quantitative analyses of images.

The need for a method to rapidly quantify the absolute number of nanoparticles internalized by cells led us to the development of a highly innovative method that integrates high resolution confocal microscopy with automatic image analysis. This method is called Particle_in_Cell-3D and was described in detail in a previous publication [5]. In this work we briefly describe Particle_in_Cell-3D and present how it was successfully applied to precisely quantify the cellular uptake of silica and ceria nanoparticles.

Silica nanoparticles have a wide range of applications such as in chemical mechanical polishing, cosmetics, food, additives to pharmaceutical drugs, and in biotechnological and biomedical fields [6–9]. Ceria nanoparticles can be also found in many applications, as in ultraviolet absorbers, automotive catalytic converters, fuel additives, and oxygen sensing [10–13]. Due to the extensive range of applications and to the potential risks of nanomaterials, a growing number of studies regarding the cytotoxicity of silica and ceria nanoparticles can be found in the literature. As regards silica nanoparticles, several investigations showed that the toxicity increases with decreasing particle sizes, increasing doses and longer exposure times [14–16]. In the case of ceria nanoparticles, very contradictory findings have been reported. On the one hand, the anti-inflammatory, antioxidant and radio-protective properties have been described as beneficial applications in nanomedicine [17–19]. On the other hand, oxidative stress and impaired cell viability were shown to be a function of the particle dose and the exposure time [1,20]. However, most of the studies concerning the interaction of silica and ceria nanoparticles with cells cannot be directly compared as they were performed by applying different cell types and a variety of different particles. Nanoparticles, such as ceria released from automotive catalytic converters, can be taken up via the respiratory tract and then be transferred into the blood stream [21]. Next, the nanoparticles will be in contact with endothelial cells lining the inner surface of our blood vessel system [22–23]. Endothelial cells play a crucial role in many physiological processes and an altered endothelial cell function can be found in innumerous diseases of the cardiovascular, pulmonary, and neurologic systems [24–25]. Therefore, endothelial cells such as the ones used in the present study (HUVEC and HMEC-1) represent a very appropriate model system to estimate the impact of nanoparticles on human health.

## Results and Discussion

### Particle_in_Cell-3D

Particle_in_Cell-3D [5] is a custom-made macro for the widely used ImageJ software [26] and can be downloaded from the ImageJ Documentation Portal [27]. It is a semi-automatic image analysis routine designed to quantify the cellular uptake of nanoparticles by processing image stacks obtained by two-color confocal fluorescence microscopy. One emission channel is reserved for the plasma membrane and the other one for the nanoparticles. This means that cell membrane and particles must be fluorescently labeled with spectrally separable markers. The two image stacks acquired can then be processed by Particle_in_Cell-3D.

**Figure 1:**
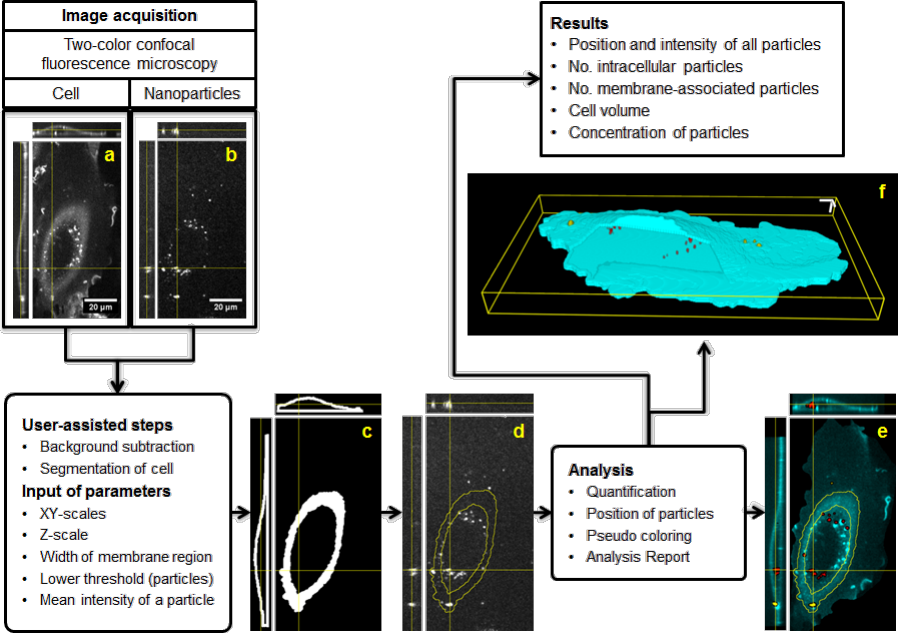
Particle_in_Cell-3D processing overview. (a) Representative confocal section image and orthogonal projections of a HUVEC cell membrane stained with CellMask™ Deep Red. (b) The respective image of silica nanoparticles labeled with perylene, a fluorescent dye. The 3D location of an intracellular particle is marked by the crossing yellow lines. (c) A smoothing filter is applied and the image of the cell is transformed into a white mask. The image stack of masks is further processed to deliver a 3D reconstruction of the cell boundaries. Intracellular and membrane region are also defined in this step. (d) The cell boundaries, or regions of interest, are then used to segment the image of the nanoparticles (yellow outline). The segmentation procedure occurs throughout the image stack, leading to a 3D localization of the particles with respect to the cell. (e) Quantitative image analysis takes place. The intensity of each object (particle or agglomerate) is compared to the intensity of a single particle previously measured in a calibration procedure. Nanoparticles are pseudo-colored according to the cellular region. In this example the cell membrane is shown in cyan, the intracellular nanoparticles appear in red, and the membrane-associated nanoparticles in yellow. (f) 3D representation of nanoparticle uptake after evaluation. Intracellular nanoparticles can be seen through the window intentionally open in the membrane region (cyan). 3D scale bars = 5 µm.

Once the images are loaded, it will execute a series of ImageJ commands to accomplish its goals. The initial part (files selection, input of analysis parameters and 3D reconstruction of the cell) are user-assisted. After these preliminary steps, automatic processing takes place (Figure 1). Particle_in_Cell-3D uses the image of the membrane to define two subcellular regions or interest: intracellular volume and membrane region. Each particle (or agglomerate of particles) is pseudo-colored according to its location and quantified according to its fluorescence intensity. A final analysis report delivers information about the position of each object, the number of nanoparticles forming that object, and its location in x,y,z coordinates. All input parameters, processed images, and results are saved and can be accessed at any time. Furthermore, as a calibration experiment is needed for measuring the fluorescent intensity of individual nanoparticles, Particle_in_Cell-3D has a routine to perform these measurements.

#### Main features

The main advantages of this method are its speed, reliability and accuracy. The complete analysis of one cell is performed in a few minutes. Moreover, the results are consistent, that is to say, Particle_in_Cell-3D substitutes the subjective character of human-assisted image analysis by its unbiased outcomes.

The cell segmentation strategy employed by Particle_in_Cell-3D includes the formation of a three-dimensional membrane region. The width of this region is set by the user and defines an enlarged transition region between extra- and intracellular spaces. It is much wider than the real cell membrane. The accuracy of the cell segmentation strategy and the typical thickness of the enlarged membrane region were studied by comparing the results achieved with Particle_in_Cell-3D with quenching experiments. It was shown that the typical width of the membrane region is about 1.4 µm and that our method is able to create a 3D reconstruction of the cell.

As regards the accuracy, the counting strategy of Particle_in_Cell-3D is based on the fluorescence intensity of the nanoparticles. The mean intensity of a single nanoparticle, obtained through a calibration experiment, is compared to the intensity of each object and determines the number of nanoparticles forming this object. It is therefore assumed that the self-quenching of dyes in particle agglomerates is negligible. This approach was proved to be accurate by independent stimulated emission depletion (STED) microscopy, a super-resolution technique [28–29].

Although developed for the absolute quantification of the nanoparticle uptake by cells, this method was made flexible to allow for the quantification in absolute and also in relative values. For example, Particle_in_Cell-3D was used to compare the uptake efficiency of therapeutic nanoparticles for gene delivery functionalized with different targeting ligands [30]. In addition, our method was successfully applied to measure the influence of flow conditions on the cellular uptake of nanoparticles. The flow is generated by a novel microfluidic reactor that can be combined with live-cell imaging and is able to cover the entire physiological range of shear rates [31].

#### Comparison to other methods

Customary techniques performed for achieving the dosage of particles taken up by cells include flow cytometry, mass spectroscopy, electron and light microscopies [32–39]. Flow cytometry provides sound statistics due to the large number of cells evaluated in a short time. Nevertheless, it does not deliver spatial information about the position of nanoparticles interacting with the cells, e.g., membrane-associated particles and intracellular particles. Mass spectroscopy offers very high sensitivity, but is a sample-destructive technique and spatial information is not obtained. Moreover, results are normally expressed in arbitrary units, and not in absolute numbers. Electron microscopy allows one to achieve detailed information with very high spatial resolution, but the price to pay is to work on fixed cells, with an elaborated sample preparation and time-consuming measurements.

Light microscopy can be used on live cells to acquire loads of data relatively fast. On the other hand, standard light microscopes such as confocal and wide-field instruments are limited by diffraction. The resolution of light microscopes is not enough to resolve particles smaller than approximately 200 nm and a direct quantification of nanoparticles is not possible. Complications to count nanoparticles are further increased by their tendency to agglomerate in biological media [40]. Our digital method was designed to circumvent the abovementioned restrictions of conventional light microscopy. It does not enable the absolute quantification of particles by overcoming the diffraction barrier, but by inferring particle numbers based on the fluorescence intensity of particles.

### Cell type-dependent uptake of silica nanoparticles

In a preceding publication [4] we found that both the uptake behavior and the cytotoxicity of silica nanoparticles are cell type-dependent, but not interconnected. In this section, we want to present in detail how Particle_in_Cell-3D was used to study the cell type-dependent uptake of 310 nm silica nanoparticles into human vascular endothelial cells (HUVEC) and cancer cells derived from the cervix carcinoma (HeLa).

The nanoparticle uptake by single cells was measured through confocal microscopy in a time series between 1 and 24 h. The concentration of nanoparticles was 39.5 µg·mL^−1^ (or 30000 nanoparticles per cell) in all experiments. We found that within the first 4 h of incubation the number of intracellular particles was up to 10 times higher for HUVEC than for HeLa cells. However, after 10 or 24 h of interaction, the amount of particles taken up by HeLa cells strikingly exceeded the amount of silica particles taken up by HUVEC cells.

#### Characterization of silica nanoparticles

In order to allow for the investigation with live-cell imaging, silica nanoparticles were labeled with perylene dye. A detailed description of the synthesis can be found in a previous publication [41]. From experiments on the labeling efficiency of perylene, it was estimated that dye molecules cover only about 0.16% of the surface of the particles and, therefore, should not influence the interaction between particles and cells. In fact, cytotoxicity measurements of labeled silica particles compared to unlabeled silica particles showed that the label did not influence the interaction between nanoparticles and cells. The size of the silica particles, 310 ± 37 nm, was determined by transmission electron microscopy (TEM). In addition, the hydrodynamic diameter of the particles over time was determined by dynamic light scattering (DLS) measurements in water and in cell medium. Depending on the properties of the nanoparticles, they may agglomerate in a given cell medium [40]. In the case at hand, the silica particles became slightly agglomerated as the mean particle size increased from 450 nm, when measured in water, up to sizes between 550 nm and 650 nm for all time points investigated. Besides the size, the zeta potential of the particles was determined to be −14.1 ± 1.5 mV in cell medium. For the quantitative evaluation with Particle_in_Cell-3D, it was necessary to measure the mean fluorescence intensity of a single silica nanoparticle. This calibration experiment was carried out by using the same microscope setup used for the cellular uptake experiments, but instead of having cells incubated with nanoparticles, the particles were deposited and spread on a cover slip and, in order to maintain the same environmental conditions, cell medium was added to the particles. The acquired images were evaluated with the subroutine ‘Calibration’ of our macro and the mean intensity showed a Gaussian distribution with a mean value of 48090 pixel intensities per nanoparticle for silica particles in the cell medium for HeLa cells and 49430 pixel intensities per nanoparticle for silica particles in the cell medium for HUVEC cells.

#### Quantification of silica-nanoparticle uptake

**Figure 2:**
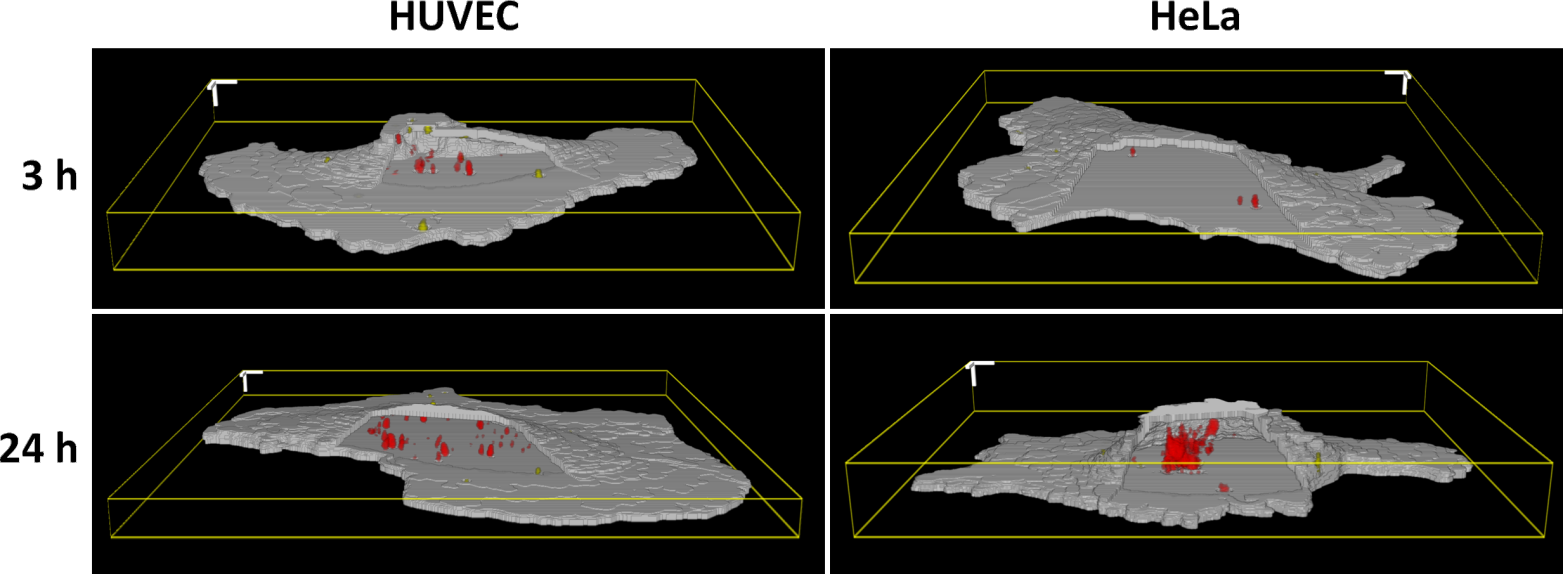
Cell type-dependent uptake kinetics of silica nanoparticles. The figure shows representative three-dimensional reconstructions of 310 nm silica nanoparticles interacting with HUVEC or HeLa cells after 3 and 24 h. The membrane region outlining the cells appears in gray. A window was purposefully open in the 3D perspectives so as to allow the visualization of intracellular particles (in red). Particles situated within the membrane region are shown in yellow. An increasing number of particles taken up in the cells over time is clearly observed for both cell types, while being much more prominent for HeLa cells. 3D scale bars = 5 µm.

In order to investigate the cell type-dependency of the uptake kinetics of silica nanoparticles, living cells were incubated for different time periods: 1, 2, 3, 4, 10 and 24 h. After incubation, the cell medium the containing nanoparticles was removed and the plasma membrane was stained. Confocal image stacks were then acquired and analyzed with Particle_in_Cell-3D. Figure 2 shows representative 3D perspectives of silica nanoparticles internalized by HUVEC and HeLa cells after 3 and 24 h. By using this method it was possible to precisely localize and quantify the particles interacting with the cells.

**Figure 3:**
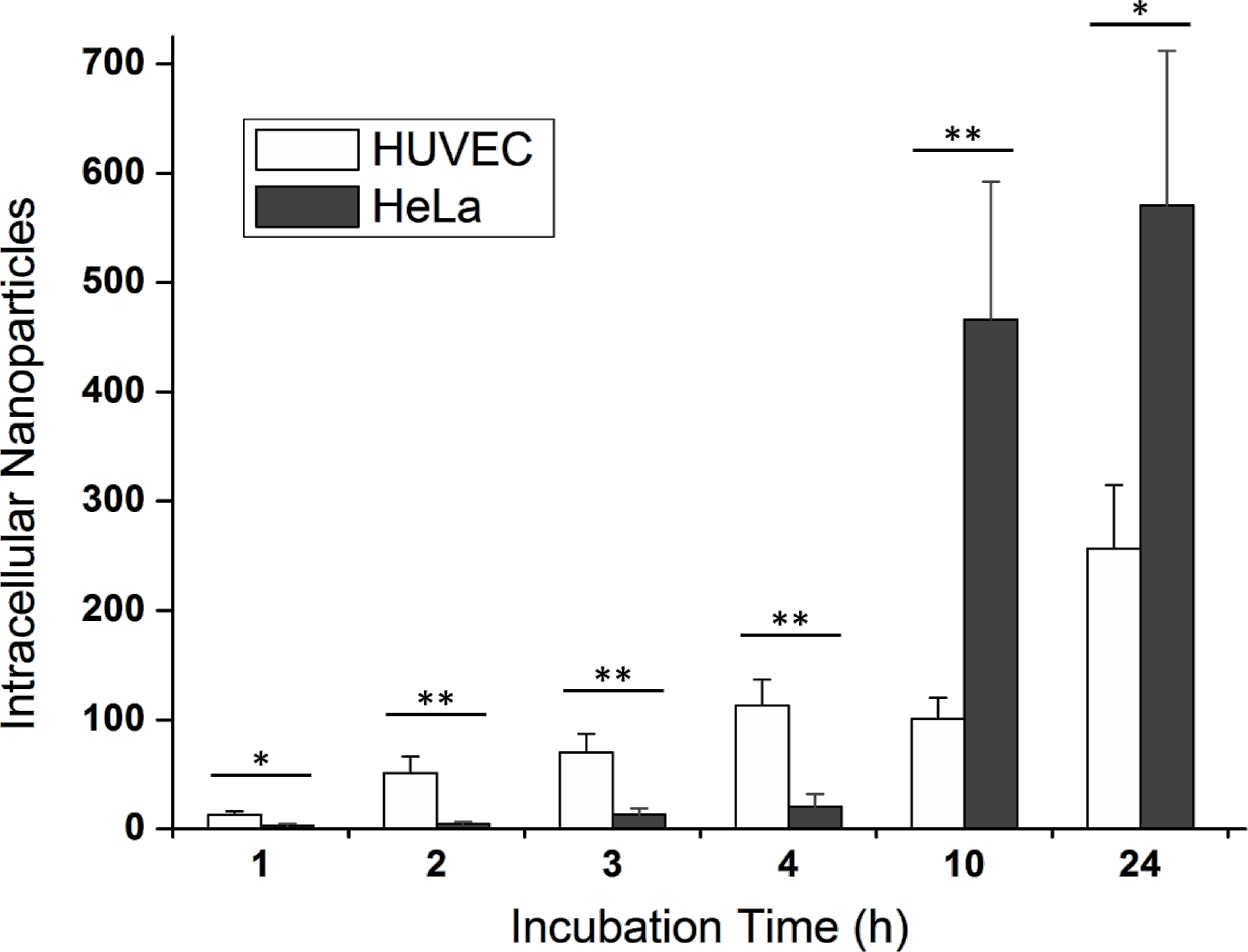
Uptake kinetics of silica nanoparticles in HUVEC (white) and HeLa cells (dark gray). During the first 4 h the mean number of incorporated nanoparticles increased linearly for both cell types. The average number reached 113 ± 24 nanoparticles for HUVEC cells and only 20 ± 11 particles for HeLa cells. Remarkably, the situation was reversed after 10 h, when a larger number of silica nanoparticles were incorporated by HeLa cells. After 24 h, the mean value of taken up particles for HUVEC cells was 256 ± 58, while for HeLa it was 570 ± 141. The histograms show the mean ± standard error of the mean of at least three independent experiments (*n* = 28–32). Results were statistically different (*p < 0.05) for incubation times of 1 and 24 h and highly statistically different (** *p* < 0.01) for all other time points.

The number of intracellular particles varied considerably from cell to cell. About 30 cells were evaluated per time point, thus resulting in more than 360 cells in total. The statistics for the number of taken up particles per HUVEC or HeLa cells are plotted in Figure 3. A time-dependent increase of nanoparticles from 1 to 24 h is clearly seen for both cell types. Interestingly, HUVEC cells were more efficient than HeLa cells to incorporate particles within the first 4 h. However, the situation changed completely after 10 or 24 h, when the number of intracellular particles for HeLa cells was significantly larger than that for HUVEC cells.

Strikingly, our results regarding the cytotoxicity of silica nanoparticles [4] did not reflect our finding for the uptake kinetics. Exposure to silica nanoparticles over 24 h induced cell death in HUVEC but not in HeLa cells. Yet, after 24 h the number of particles internalized by HeLa cells was twice as large as the number of particles incorporated by HUVEC cells. Quantitative determination of nanoparticle uptake with Particle_in_Cell-3D helped to show that the nanotoxicity of materials cannot be generalized and transferred from one cell type to another.

### Size-dependent uptake kinetics of ceria nanoparticles

This section is devoted to present quantitative results on the particle size-dependent uptake kinetics of ceria nanoparticles of 8 nm and 30 nm. A massive agglomeration of nanoparticles in cell medium was found. Ceria nanoparticles of 8 nm and 30 nm clustered into 417 nm and 316 nm agglomerates, respectively. Nanoparticles at a concentration of 10 µg·mL^−1^ were incubated with human microvascular endothelial cells (HMEC-1) for 3, 24, 48 and 72 h and imaged through live-cell confocal microscopy. Cytotoxicity assays performed on similar nanoparticles have shown that, in general, the impact of ceria nanoparticles on endothelial cells (HUVEC and HMEC-1) is not significant, and that adverse effects can only be observed at concentrations as high as 100 µg·mL^−1^ [42]. Such doses exceed the maximum possible in vivo concentrations.

#### Characterization of ceria nanoparticles

In order to be investigated with fluorescence microscopy, the particles were marked with Atto 647N. The synthesis of the ceria nanoparticles investigated in this study is described in the literature [43]. The labeling of these particles with Atto 647N did not alter the biological response of the cells, as assessed by cytotoxicity assays. HMEC-1 cells were incubated over 48 h with 100 µg·mL^−1^ of either non-labeled or Atto 647N-labeled ceria nanoparticles. After this period, the relative adenosine triphosphate (rATP) content was analyzed to determine the metabolic impact of nanoparticles on cells. One hundred percent rATP content would mean that the cellular viability of the cells treated with nanoparticles matches the viability of untreated cells. As shown by Strobel et al. [42], incubation with non-labeled 8 nm and 30 nm ceria nanoparticles resulted in rATP values (mean ± standard deviation) of 82.0 ± 5.6% and 76.3 ± 10.8%, respectively. The rATP contents measured after the exposure to Atto 647N-labeled nanoparticles of 8 nm and 30 nm were 80.1 ± 6.2% and 79.5 ± 14.9%, respectively. Therefore, the fluorescent labeling of the ceria nanoparticles presented in this work did not significantly alter the cytotoxicity of these particles on HMEC-1 cells. The primary size of the two nanoparticles was determined through TEM. One particle type has a diameter of 8 nm and is spherical (CeO_2_-8nm), while the other particle type has a diameter of roughly 30 nm (CeO_2_-30nm) (ellipsoid of 27 nm × 30 nm). It has been shown that the smaller the nanoparticles, the stronger the agglomeration [40]. This has been confirmed in the determination of the hydrodynamic diameter of these particles. DLS measurements were carried out and the size of CeO_2_-8nm increased up to 417 nm in cell medium. In the case of the CeO_2_-30nm particles, the diameter in cell medium was determined to be 316 nm. The zeta potential was also assessed in cell medium: −11.3 mV for the 8 nm particles and −12.3 mV for the 30 nm particles.

The same procedure described for the silica nanoparticles in the previous section was used to measure the mean fluorescence of single ceria particles. The results were intensities of 131201 pixels (CeO_2_-8nm) and 742814 pixels (CeO_2_-30nm), respectively. There is an important particularity to be mentioned here. The mean intensity of the single particles is in fact the mean intensity of single agglomerates, as it was not possible to obtain single nanoparticles of primary sizes for the calibration experiments. Those agglomerates, however, are in fact the particles that interact with the cells.

#### Quantification of ceria nanoparticle uptake

**Figure 4:**
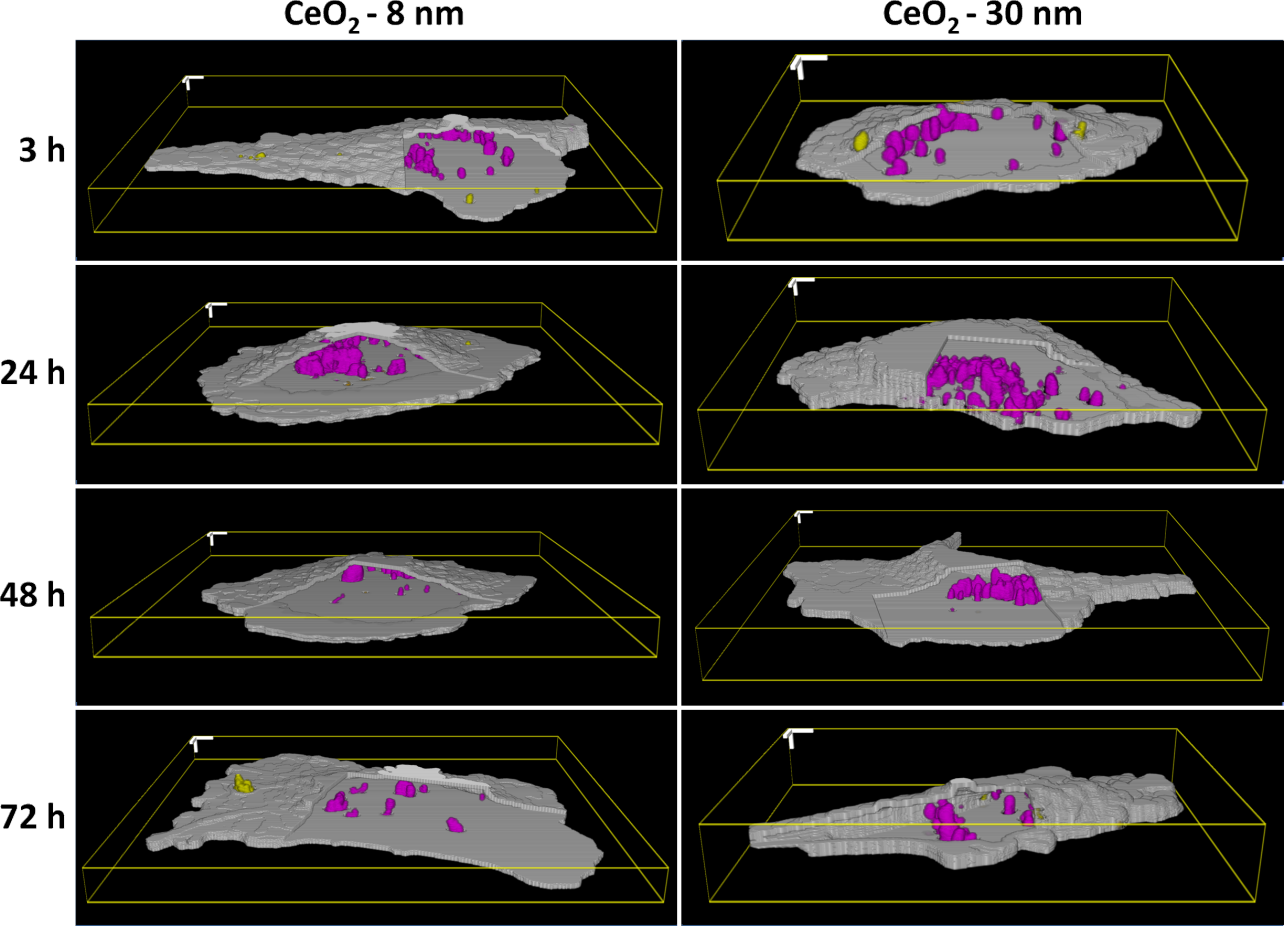
Particle size-dependent uptake kinetics of ceria nanoparticles by HMEC-1 cells. Representative three-dimensional images of 8 nm and 30 nm ceria nanoparticles interacting with endothelial cells for 3, 24, 48 and 72 h are shown. Due to the strong agglomeration of particles, the individual particles quantified by Particle_in_Cell-3D are actually individual agglomerates of 417 nm (CeO_2_-8 nm) and 316 nm (CeO_2_-30nm). The membrane region outlining the cells appears in gray. The intracellular nanoparticles can be visualized in magenta and particles interacting with the membrane appear in yellow. The agglomerates are taken up by cells inside endosomes and accumulate at the perinuclear region. The amount of internalized particles is increasing over 24 h, but after this incubation time, however, the number of particles inside the cells starts to decrease. This effect is more remarkable for the 8 nm nanoparticles than for the 30 nm nanoparticles. 3D scale bars = 5 µm.

With the purpose of investigating the size-dependent uptake kinetics of ceria nanoparticles for a longer time than traditionally, HMEC-1 cells were incubated with 8 nm (417 nm) and 30 nm (316 nm) nanoparticles for 3, 24, 48 and 72 h. Figure 4 presents illustrative images of the interaction of ceria nanoparticles with endothelial cells.

**Figure 5:**
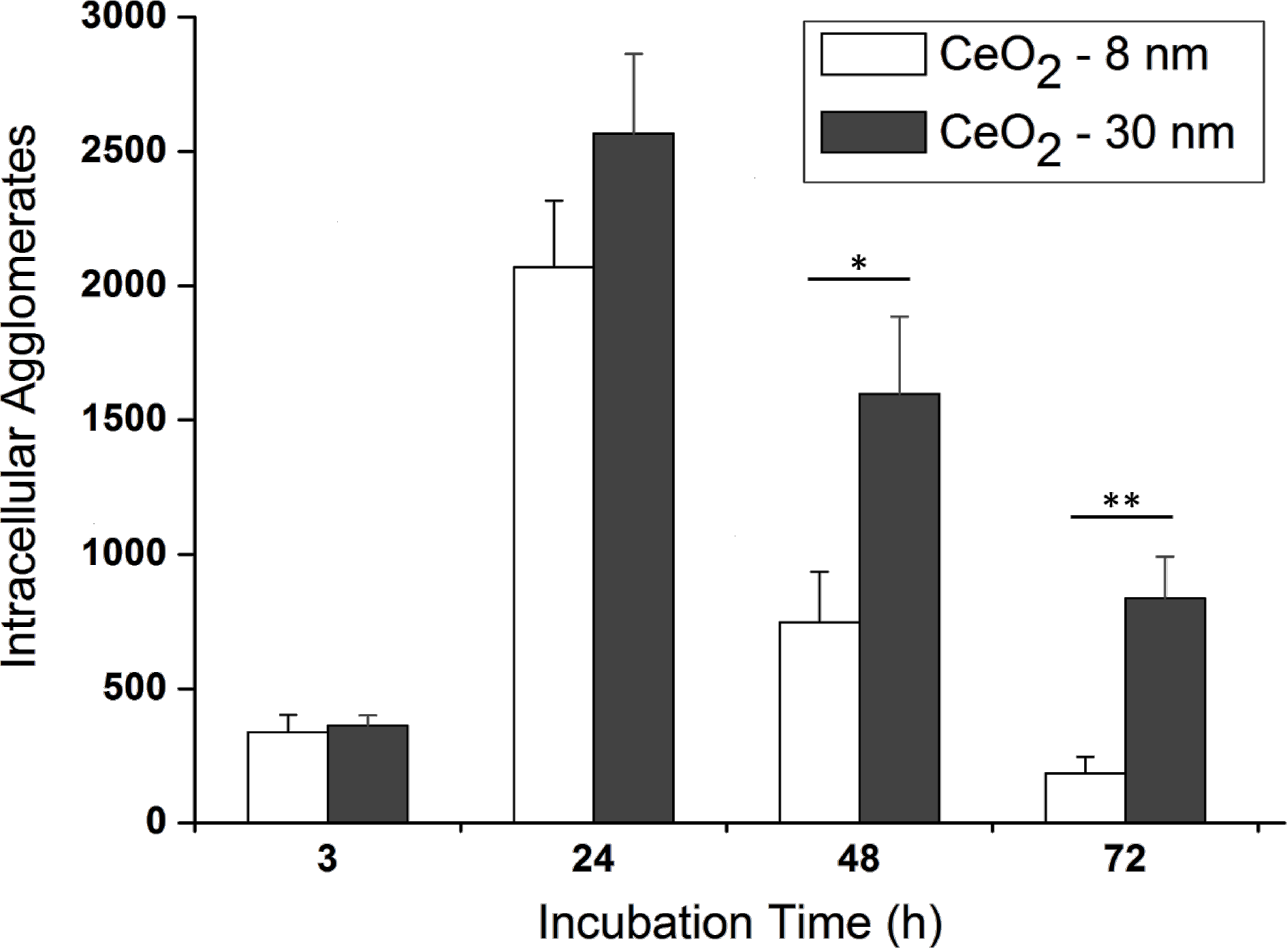
Uptake kinetics of 8 nm (white) and 30 nm (dark gray) ceria nanoparticles in HMEC-1 cells. The number of internalized nanoparticles after 3 h is practically the same for both particle sizes. These numbers then escalates to reach a maximum at around 24 h. After 48 and 72 h, however, the number of particles incorporated by the cells is reduced back to amounts similar to that measured after 3 h. The histograms show the mean ± standard error of the mean of at least two independent experiments (*n* = 12–16). Results were statistically different (*p < 0.05) for an incubation time of 48 h and highly statistically different (**p < 0.01) for 72 h.

Approximately 15 single cells were measured per time point and per particle type, resulting in a total of 115 cells analyzed in great detail by Particle_in_Cell-3D. These quantitative results are presented in Figure 5 and show that the number of incorporated particles increases steeply between 3 and 24 h, with no significant difference between the two particle sizes. The number of internalized agglomerates of CeO_2_-8nm nanoparticles increased from 337 ± 66 to 2069 ± 248, whereas it increased from 363 ± 37 to 2567 ± 297 for CeO_2_-30nm agglomerates. After this point in time, however, the number of intracellular particles is decreased back to initial levels, 185 ± 61 agglomerates for CeO_2_-8nm and 836 ± 155 for CeO_2_-30nm particles. The dilution of intracellular nanoparticles has been shown to be caused by cell division, as reported in a recent publication [44]. As cells undergo mitosis, intracellular particles of the mother cells are shared with the daughter ones. Cell division may therefore have direct influence by decreasing the number of taken up particles with time. Since the doubling time of HMEC-1 cells is 28.6 h [45], and the dilution of intracellular ceria nanoparticles occurs after 24 h, cell division probably plays an important role in our findings.

Cell division is probably among the dominant causes for the observed dilution of nanoparticles. Yet, other time-dependent parameters may also influence the uptake dynamics. For example, degradation of intracellular particles, exocytosis, cell uptake behavior (e.g., cell-cycle phase dependency, and load capacity), and the number of nanoparticles available for uptake.

## Conclusion

The possibility to quantify nanoparticles on the single-cell level is an important step to better understand the mechanisms of nanoparticles-cell interactions. In this work it was demonstrated that results achieved with Particle_in_Cell-3D were decisive to determine the cell type-dependent uptake kinetics of silica nanoparticles. Moreover, the quantification of intracellular ceria nanoparticles showed that there is a significant difference in the uptake kinetics of 8 nm (agglomerate size 417 nm) and 30 nm (agglomerate size 316 nm) nanoparticles. After 48 h, the particles that form smaller agglomerates, i.e., 30 nm nanoparticles, are internalized more efficiently by endothelial cells. In addition, our findings offered a new insight into the remarkable dilution of intracellular nanoparticles, possibly influenced by cell division.

Particle_in_ell-3D can be applied to investigate the dose-dependent effects for the risk assessment of nanoparticles. Additionally, this method can be used to study which factors are determinant for the successful attachment, internalization and cargo release of nanoparticles designed for medical applications.

## Experimental

### Nanoparticle characterization

Nanoparticle size was determined by transmission electron microscopy (TEM). TEM micrographs were acquired by a JEM 2011 (JEOL, Japan) transmission electron microscope. The nanoparticle dispersion was diluted with EtOH or MeOH and applied onto a carbon-coated copper grid (Plano, Formvar coal-film on 200 mesh-net). The sizes of the nanoparticles were then determined from TEM images through digital image analysis with the ImageJ software [26].

Zeta potentials and hydrodynamic diameter (through dynamic light scattering) were measured in ultrapure water and in cell medium (see section ‘Cell culture’ for details) with a Zetasizer Nano (Malvern Instruments, UK). In order to break down agglomerates, the resulting solution was vortexed for 10 s, treated in an ultrasonic bath for 10 min and vortexed again for 10 s.

### Cell culture

HeLa and HUVEC cells were grown as described previously [4]. HMEC-1 cells were grown in MCDB-131 medium (Life Technologies, Germany) supplemented with 10% fetal bovine serum (Life Technologies), 1% Glutamax (Life Technologies), 10 ng·mL^−1^ human epidermal growth factor (Life Technologies) and 1 µg·mL^−1^ hydrocortisone (Sigma-Aldrich). Cells were kept in a humidified 5% CO_2_ atmosphere at 37 °C.

#### Uptake experiments

For live-cell imaging experiments, cells were seeded 24 h before imaging in 8-well Nunc™ Lab-Tek™ II chamber slides (Thermo Fisher Scientific Inc., Germany) at a density of 1.1 × 10^4^ cells·cm^−2^. HeLa and HUVEC cells were incubated with silica nanoparticles as described before [4]. HMEC-1 cells were incubated with ceria nanoparticles in humidified 5% CO_2_ atmosphere at 37 °C. The 10 µg·mL^−1^ solution of ceria nanoparticles was prepared in the same cell medium used for cell growth. Before addition to cells, the solution was vortexed for 10 s, treated in an ultrasonic bath for 10 min and vortexed again for 10 s. After the incubation time, and just before measurements, the cell membrane was stained with a solution of 10 µg·mL^−1^ wheat germ agglutinin, Alexa Fluor® 488 (Life Technologies) in cell medium, incubated at 37 °C for 1 min, and washed twice with warm cell medium.

#### Cytotoxicity assay

The procedure for the determination of the relative cellular ATP level of ceria nanoparticles is described in detail by Strobel et al. [42].

#### Live-cell imaging

Imaging was performed on a Zeiss spinning disk confocal fluorescence microscope equipped with a Zeiss Plan Apochromat 63× /1.40 Oil/DIC objective. Samples were in 5% CO_2_ atmosphere at 37 °C during imaging and were illuminated with laser light alternating between 488 nm and 639 nm, exciting the cell membrane stain and the Atto 647N dye (labeling the ceria nanoparticles), respectively. Image sequences were captured with an electron multiplier charge-coupled device camera (Evolve 512, Photometrics, USA). Several planes of the cells were imaged with a spacing of 250 nm and a detection time of 100 ms per confocal section.

#### Statistics

The unpaired Student’s t-test was used for statistical analyses. Values were expressed as the mean ± standard error of the mean. Results were considered to be statistically different at *p* < 0.05 and highly statistically different at *p* < 0.01. For the determination of the relative ATP content, values represent the means ± standard deviation (*n* = 3).
